# Lipoproteins Are Potent Activators of Nuclear Factor Kappa B in Mammary Epithelial Cells and Virulence Factors in *Mycoplasma bovis* Mastitis

**DOI:** 10.3390/microorganisms10112209

**Published:** 2022-11-08

**Authors:** Peleg Schneider, Re’ella Brill, Iftach Schouten, Einat Nissim-Eliraz, Inna Lysnyansky, Nahum Yehuda Shpigel

**Affiliations:** 1 The Koret School of Veterinary Medicine, The Robert H. Smith Faculty of Agriculture, Food and Environment, The Hebrew University of Jerusalem, Rehovot 76100, Israel; 2 Mycoplasma Unit, Kimron Veterinary Institute, Bet Dagan 50250, Israel

**Keywords:** *Mycoplasma bovis*, lipoproteins, mastitis, murine model, immunomodulation

## Abstract

Mastitis due to *Mycoplasma bovis* is a worldwide problem, which leads to significant economic losses and affects animal welfare. However, the mechanisms by which *M. bovis* establishes and maintains intra-mammary infections (IMI) in dairy cows are largely unknown. To study in further detail the pathogenesis of *M. bovis* IMI, time- and cost-effective experimental models are needed. To this end, we established and characterized an in vitro murine mammary alveolar epithelial (EpH4) cell-based model and an in vivo murine mastitis model. Our results showed that live and UV-treated *M. bovis* field strain 161791 and its lipid-associated membrane proteins (LAMP) activated nuclear factor kappa B (NF-kB) in EpH4 cells in a dose-dependent manner. In the murine mastitis model, temporal and spatial dynamics of inflammation in the mammary tissues were evident. Live *M. bovis* elicited diffuse inflammation affecting the whole challenged gland peaking at 48 h post infection (pi) in contrast to LAMP challenge, which elicited only focal inflammation peaking at 24 h and resolving at 48 h pi. Inflammation was characterized by massive neutrophil recruitment into the milk spaces and by elevated expression of the inflammatory mediators TNF-α, KC, iNOS and NF-kB dependent genes: A20 and IkBα. Moreover, the presence of intraepithelial bacterial communities in glands challenged with live *M. bovis* bacteria was shown. The developed models can be used efficiently for future characterization of *M. bovis* virulence factors and host immune response to IMI.

## 1. Introduction

*Mycoplasma bovis* is one of the major contagious mastitis pathogens causing clinical, subclinical, and chronic intramammary infection (IMI), which leads to significant economic losses and affects animal welfare [1,2,3,4]. Traditional biosecurity measures that have effectively reduced the prevalence of most contagious mastitis pathogens [5] are either partially effective or ineffective for mycoplasma mastitis. Moreover, the current lack of efficacious antibiotics or vaccines to treat or prevent *M. bovis* mastitis renders animal culling the recommended practice for controlling this disease [2].

The successful establishment and persistence of an IMI are governed by bacterial virulence factors as well as by the nature of the host immune response elicited by the invading pathogen [6]. The current knowledge regarding microbial characteristics and host–pathogen interactions that allow *M. bovis* to impair the host immune system and to advance an infection has been recently reviewed [7,8]. The consequences of *M. bovis* interaction with the immune system include the ability to evade opsonization and to impair effective phagocytosis by neutrophils and macrophages, neutrophil activities modification, and neutrophil extracellular traps (NETs) degradation (reviewed in [7,8]). In addition, *M. bovis* impacts the cytokine production of different types of cells, influences programmed cell death through delayed monocyte/macrophage apoptosis, suppresses lymphocyte activities such as Th1 cytokine production and induces lymphocyte apoptosis (reviewed in [7,8]). Moreover, the suppression of the immune response, so-called T-cell exhaustion, caused through the PD-1/PD-L1 pathway has been demonstrated in cattle infected with *M. bovis* [9,10,11,12]. Such T cell dysfunction that arises during many chronic infections as well as in cancer might lead to increased susceptibility to other infections and to exacerbation of disease associated with co-infection [13]. *M. bovis* also successfully invades and survives inside a variety of bovine cells such as alveolar macrophages, lymphocytes, and erythrocytes, as well as within different types of epithelial cells including bovine mammary epithelial cell (bMEC) [14,15,16,17]. Despite the above-mentioned knowledge, the mechanisms by which *M. bovis* IMI is established and maintained in dairy cows as well as the nature of the innate immune response (IIR) to *M. bovis* mastitis are still largely unknown.

Innate immunity plays an important role in mastitis, serving as the first line of host defense and dictating the outcome of IMI [6,18]. The innate immune system differentially elicited inflammatory responses are mediated through ligand-specific activation of host pattern recognition receptors (PRRs), among which the Toll-like receptors (TLRs) are the most common ones; the TLRs recognize distinct molecular motifs, commonly referred to as microbe-associated molecular patterns (MAMPs) [19,20,21]. For example, TLR2, in concert with other TLR family members, recognizes peptidoglycan and lipoteichoic acid from Gram-positive bacteria [22,23,24] as well as lipopeptides from *Mycoplasma* spp. including *M. bovis* [25,26,27,28,29,30,31,32], while TLR4 recognizes lipopolysaccharide (LPS) from Gram-negative bacteria [33]. Moreover, several studies reported that in addition to TLR2, *M. bovis* may activate additional TLRs on bMECs membrane surface including TLR4 [34], TLR1 and TLR6 as well as cytoplasmatic TLR3 and TLR9 [35].

The activation of TLRs leads to production of cytokines, chemokines, and activation of nuclear factor kappa B (NF-κB), the master regulator of inflammation and immunity in all body tissues including the mammary gland [36,37,38]. The activation of NF-κB by *Mycoplasma* spp. and their lipid-associated membrane proteins (LAMPs) has been previously shown [27,28,29,30,31,32,35,39]. In addition, differentially expressed long non-coding RNAs (lncRNAs) related to the NF-κB signaling pathway were recently identified in bovine mammary gland tissues infected with *M. bovis* [40]. Eventually, activation of cytokines and NF-κB results in massive recruitment of neutrophils into the alveolar milk space; these neutrophils serve as the main protective cells in mastitis and their recruitment is the hallmark immune response of mastitis [41,42].

Studying *M. bovis* virulence factors and the immune response caused by *M. bovis* in the host is partially hindered due to a lack of appropriate genetic tools as well as suitable, time- and cost-effective experimental models allowing high throughput screening and systematic analysis of this pathogen. However, recent progress made in the development of molecular tools facilitating the genetic manipulation of animal mycoplasmas [43,44,45,46,47] offers new horizons for the investigation of *M. bovis* pathogenesis, although in vitro and in vivo experimental models are still needed. Therefore, to further investigate the pathogenesis of *M. bovis*, we established and characterized in vitro murine mammary alveolar epithelial (EpH4) cell-based and in vivo murine mastitis model systems. We analyzed if *M. bovis* activates NF-κB in EpH4 cells and whether there is a difference in NF-κB activation upon the challenge of EpH4 cells with different derivatives of *M. bovis* such as live, UV-treated cells and *M. bovis*-LAMPs. In the murine mastitis model, lactating mice were challenged by intra-mammary injection via the teat canal with live *M. bovis* bacteria or with LAMPs. Mammary inflammation was analyzed using intravital whole-body imaging, histological analysis of harvested mammary tissues, and qPCR to test the expression of inflammatory mediators. Furthermore, the temporal and spatial dynamics of inflammation in mice mammary tissues were evaluated.

## 2. Materials and Methods

### 2.1. Ethics Statement

All mice were maintained under specific pathogen-free (SPF) conditions and handled under protocols approved by the Hebrew University Animal Care Committee, according to international guidelines. IACUC approvals were obtained prospectively (Ethics Committee for Animal Experimentation, the Hebrew University of Jerusalem; MD-14-14318-3).

### 2.2. M. bovis Strain, Cell Lines, and Culture Conditions

*M. bovis* field strain 161791, isolated from a milk sample taken from a cow with clinical mastitis in Israel in 2012 [48]. The genotypic profile (sequence type; ST) of the isolate has been assigned as ST33 using the revised multilocus sequence typing (MLST; https://pubmlst.org/; accessed on 1 July 2022) as previously described [49]. Moreover, phylogeny based on the results of whole-genome-single nucleotide polymorphism analysis placed *M. bovis* 161791 within the clade mostly consists of isolates related to the dominant Israeli mastitis-associated genotype (ST52) [48]. *M. bovis* ST33 were also isolated from womb of cows and from calves with arthritis present on the same farm from which *M. bovis* 161791 has been isolated as well as from the cases of clinical mastitis on several other dairy farms.

*M. bovis* was propagated at 37 °C in FF-modified broth medium [50] supplemented with 0.5% (*w*/*v*) sodium pyruvate and 0.005% (*w*/*v*) phenol red. Stock cultures were grown to the titers of 10^8^–10^9^ color forming units (CFU)/mL, aliquoted, and maintained at −80 °C. For each stock, the number of CFU per ml was determined by performing serial 10-fold dilutions in FF broth and by plating each dilution on agar in triplicates [51]; the agar plates were grown at 37 °C, under an atmosphere of 5% CO_2_/95% air.

Murine mammary epithelial cell (mMEC) line EpH4 was cultured as previously reported [52,53] and the mycoplasma-free status of the cells was tested by using a genus-specific PCR [54]. To test activation of EpH4 via NF-kB the cells were infected with the lentiviral NF-kB luminescence reporter system (EpH4/NF-κB transduced cells) as previously described by us [53].

### 2.3. Inactivation of M. bovis by UV-Irradiation

*M. bovis* strain 161791 was cultured in FF medium and incubated at 37 °C until mid-log phase. Mycoplasmas cells were harvested by centrifugation at 12,000× *g* for 30 min, washed twice with phosphate-buffered saline (PBS; Biological Industries, Beit Haemek Ltd., Beit Haemek, Israel), and resuspended in PBS. An amount of 300 μL of mycoplasma cells (final concentration 10^7^ cfu) was irradiated using Stratalinker^®^ UV Crosslinker Model 1800 (Stratagene, CA, USA) for 30 min. The titer of the cell cultures before and after irradiation was determined by 10-fold serial dilutions plated on FF agar. The treated cells were stored at 4 °C for the entire period of experiment.

### 2.4. Activation of EpH4 Cells

Approximately 1 × 10^5^ EpH4 cells/well, transduced with luciferase gene under the control of 5 copies of the NF-κB response element, were infected with either live *M. bovis* 161791 (MB) and *Escherichia coli* P4 (ECP4) at a multiplicity of infection (MOI) 1:1–1:1000, with UV-treated *M. bovis* 161791 cells (MB-UV; MOI of 1:10–1:1000), or with *M. bovis*-LAMPs at concentrations 0.25, 0.5, 1 and 2 μg/mL. NF-κB activation was determined via luciferase activity, measured using 150 µg/mL luciferase substrate D-luciferin potassium salt (GoldBio), added after infection of EpH4. The luminescence was measured every 5 min for 3 h and expressed as photons/second. In all experiments, 1 µg/mL *E. coli* LPS and RPMI medium (Sigma-Aldrich, Rehovot, Israel) were used as positive and negative controls, respectively.

### 2.5. M. bovis Intramammary Challenge of Lactating Mice

Mycoplasma cultures were grown until mid-log phase and then harvested by centrifugation at 10,000× *g* for 10 min at 4 °C. The bacterial pellets were washed twice with the same volume of sterile PBS × 1 and centrifuged as described above. The bacterial cultures were resuspended in 500 µL of PBS to a final concentration of ≈2 × 10^10^ CFUs/mL. The titer of *M. bovis* was calculated by plating serial 10-fold dilutions as described above.

Six- to eight-week-old, 7–10 days post-partum lactating C3H/HeN mice (Harlan, Jerusalem, Israel) were used for intra-mammary (IMM) challenge using *M. bovis* field strain 161791. IMM challenge was performed as previously described [41,42,55]. Three mice/six glands were used for injection in each experiment, while uninfected glands were used as a negative control. Briefly, mice pups were removed 1 h before the challenge, mice were anesthetized (1.5–2.5% isoflurane in O_2_) and the abdominal surface was disinfected with 70% ethanol and dried aseptically [41,55]. IMM challenge was performed under a binocular, using 0.3 mL insulin syringes with a 33-gauge blunt needle. The abdominal glands L4 and R4 were injected with 50 μL of bacterial suspension (≈10^9^ cfu) of *M. bovis*, or 10 μg LAMPs, LPS, or Pam_3_CSK_4_ (PAM3). After inoculation, the abdomen was disinfected again, and the mice were left to recover under heating for 2 h. All mice were sacrificed 24 h or 48 h pi and mammary tissues were bisected for histology and total RNA extraction (see below).

### 2.6. Bioluminescence and Fluorescence Imaging

In vivo imaging of animals was performed at 0 h, 24 h, and 48 h pi as previously described [53]. Briefly, hair was shaved and depilated (Nair hair removal cream) from the ventral side of the animals. Mice were injected intraperitoneally with 200 mg/kg luminol (Santa Cruz Biotechnology, Dallas, TX, USA) 10 min before bioluminescence imaging of neutrophil recruitment [56]. The mice were anesthetized (1.5–2.5% isofluorane in O_2_) and a whole-body image was acquired using IVIS Lumina Series III (PerkinElmer Inc., Waltham, MA, USA) with excitation/emission filters blocked/open for luminescence (luminol) and 460/520 for milk auto-fluorescence. Each image was displayed as a false-color photon-count image superimposed on a grayscale anatomic image. Regions of interest (ROI) were drawn on the ventral side of the animal around the mammary glands and quantified with living Image Software version 4.4 (Caliper LifeSciences, Waltham, MA, USA). Counts in the ROI were detected by CCD camera digitizer and were converted to physical units of radiance in photons/s/cm^2^/steradian.

### 2.7. Histopathological Analysis

Mice were sacrificed 24 or 48 h pi and mammary tissues were bisected for histology and total RNA extraction. Samples for histopathological analysis were fixed overnight in neutral buffered 4% PFA, at 4 °C and embedded in paraffin, and sections were cut at a thickness of 5 µm and stained with hematoxylin and eosin (H&E) according to standard procedures. For fluorescence staining, mammary tissues were fixed in 4% PFA overnight at room temperature and incubated with 15% (*w*/*v*) sucrose in PBS for 72 h at 4 °C. The 13-µm cryosections were stained with 1 µg/mL phalloidin-iFlour 647 (Abcam) and 25 µg/mL DAPI (Sigma-Aldrich, Rehovot, Israel). *M. bovis* cells were visualized using polyclonal-anti *M. bovis* antibody (Mycoplasma collection, University of Florida, Gainesville, FL, USA) and rabbit anti-goat IgG (Sigma-Aldrich, Rehovot, Israel) applied as the secondary antibody. The mammary glands were mounted with Vectashield mounting medium (Vector Laboratories, Burlingame, CA, USA) and imaged with an Axio Imager M1 208 upright fluorescence microscope (Zeiss, Germany). Confocal images were acquired using a Leica TCS SP2 laser scanning spectrum confocal system (Leica Microsystems, Wetzlar, Germany) and images were merged using Leica confocal software.

### 2.8. Mammary Gene Expression Analysis

Total RNA was isolated from challenged mammary tissue using the GeneElute Mammalian Total RNA Miniprep Kit combined with on-Column DNase I Digestion Set (Sigma-Aldrich, Rehovot, Israel). Reverse transcription of tumor necrosis factor-alpha (TNF-α), keratinocyte chemoattractant (KC); inducible nitric oxide synthase (iNOS); A20 and inhibitor kappa B alpha (IkBα) was performed using qScript cDNA Synthesis Kit (Quanta BioSciences, Gaithersburg, MD, USA), and cDNA was used for subsequent real-time PCR reactions. RT-qPCR was conducted on a StepOne Plus PCR instrument (Applied Biosystems, Waltham, MA, USA) using the oligonucleotides and FAST qPCR Universal Master Mix (Kappa Biosystems, Boston, MA, USA) as previously described [53,57]. All reactions were performed in triplicates, and the gene expression levels for each amplicon were calculated using the ΔΔCT method [58]; first gene expression level was normalized against the actin housekeeping gene; second gene expression level was normalized against unchallenged mammary glands used as negative controls (*n* = 6). Melting curve analysis was performed on each primer set to confirm amplification of a single product.

### 2.9. Statistical Analysis

All experiments were performed at least three times and representative images were chosen for publication. Relative gene expression levels were calculated by comparing experimental groups using non-parametric Mann–Whitney two independent samples test. Values were first subjected to a square root transformation and did not follow a normal distribution; thus, non-parametric statistics were used. All statistical analyses were performed using GraphPad Prism 9.1.2 (GraphPad Software, Inc., San Diego, CA, USA), and a *p* value of 0.05 or less (*) was considered significant. Data are representative of ≥6 challenged or control glands.

## 3. Results

### 3.1. Differential Activation of NF-kB in mMECs

**Figure 1:**
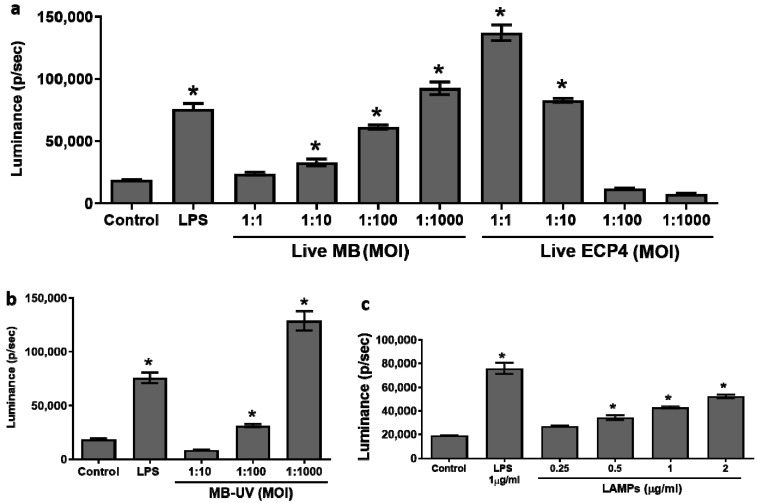
Activation of mMECs. EpH4/NF-κB transduced cells were treated with different MOIs of live *M. bovis* (MB) and *E. coli* (ECP4) (**a**), UV-treated *M. bovis* (MB-UV) (**b**) and increasing concentrations of *M. bovis*-LAMPs (**c**). NF-kB activation was measured via luciferase activity expressed as photons/second. Data are mean ± SE for one experiment performed in triplicate that represent the results of the three similar experiments. * in (**a**–**c**) indicates statistical significance in comparison with a control group (*p* < 0.05).

The capacity of different *M. bovis* preparations to activate NF-κB in mMECs was evaluated using EpH4 cells transduced with an NF-κB luminescence reporter system (EpH4/NF-κB). Significant and dose-dependent activation of NF-κB in EpH4 cells, measured via luciferase activity, was observed following exposure to live, UV-treated *M. bovis* or LAMPs (Figure 1). In comparison to live *M. bovis* (1:10), ten times the amount of UV-*M. bovis* (MOI 1:100) was needed to activate EpH4 cells (Figure 1). The NF-κB activation by live *M. bovis* differed from the activation obtained with a live *E. coli* strain P4. Indeed, at MOIs ≥ 1:10 of *E. coli*, the activation of EpH4 cells has drastically decreased probably as a result of the toxic effect of the high bacterial challenge (Figure 1a).

### 3.2. The Establishment and Characterization of M. bovis Murine Mastitis Model

**Figure 2:**
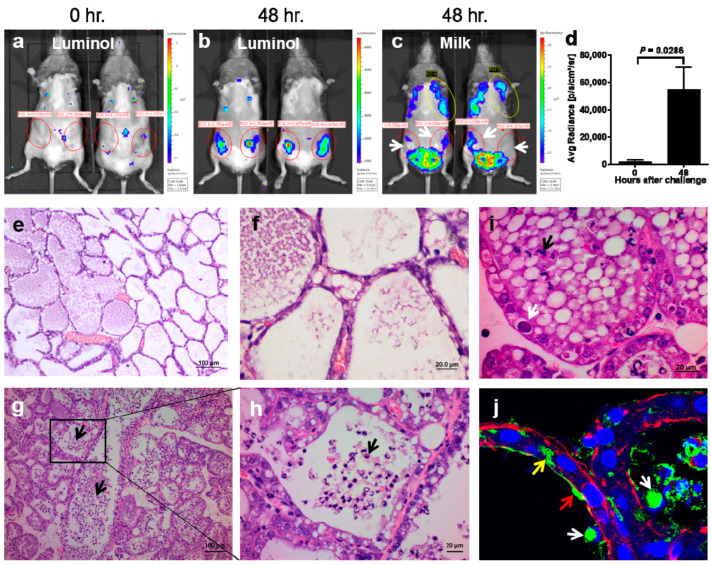
Characterization of the murine *M. bovis* strain 161791 mastitis model. Mammary virulence of *M. bovis* was demonstrated in lactating C3H/HeN mice following IMM challenge of L4 and R4 glands. (**a**,**b**) Bioluminescence imaging of neutrophil recruitment and (**d**) difference in mammary inflammation represented by luminescence intensity measured at time 0 and 48 h after challenge. (**c**) The effect of disease on milk quantity in the glands. Loss of milk in the affected glands (red circle) in comparison to non-challenged glands (yellow circle) is shown by white arrows. (**e**–**i**) H&E staining of tissue samples of normal control and challenged glands. (**e**,**f**) The control mammary gland is normal and free of disease signs, while massive neutrophil recruitment is visible in challenged glands (black arrows in (**g**–**i**)). Large colonies of *M. bovis*, identified in mMECs, are marked by white arrow (**i**). (**j**) Cryosections of mammary tissue were stained with phalloidin (red), DAPI (blue), and polyclonal-anti *M. bovis* antibody (green) and analyzed using confocal microscopy showing a single Z-stack. Microscopic image demonstrates *M. bovis* microbial communities adherent to the apical membrane of mammary alveolar epithelium (red arrow in (**j**)), intraepithelial *M. bovis* communities (yellow arrow in (**j**)), and *M. bovis* bacteria phagocytosed by alveolar neutrophils (white arrows in (**j**)). Scale bars; 100 µm (**e**,**g**) and 20 µm (**f**,**h**,**i**). Image (**i**) original magnification ×40. Representative images of ≥6 mice.

To study the interaction of *M. bovis* with the mammary gland and to understand the immune defense mechanisms of the mammary gland against this pathogen, we established a murine mastitis model. Mice were challenged IMM with ≈10^9^ CFUs of *M. bovis* 161791 as described in the Materials and Methods section. No death or external signs of disease were observed in mice up to 48 h pi. However, results of intra-vital whole-body imaging showed that in comparison to the non-injected glands, *M. bovis* challenged glands demonstrated intra-mammary inflammation characterized by intense neutrophil recruitment (Figure 2a,b), which was significantly higher (*p* = 0.0286) at 48 h pi than at time 0 (Figure 2d). In addition, the decrease in milk production, visible by milk auto-fluorescence, was evident at 48 h pi (Figure 2c).

Mammary inflammation, demonstrated by whole-body imaging (Figure 2a,b), was also supported by histological microscopic analysis of mammary tissue sections harvested 48 h pi revealing massive neutrophil recruitment into alveolar and ductal milk spaces (Figure 2g–j). Notably, large colonies of intraepithelial bacteria, visible in the H&E-stained tissue sections (Figure 2i), were further identified as *M. bovis* organisms using immunofluorescence staining (Figure 2j).

**Figure 3:**
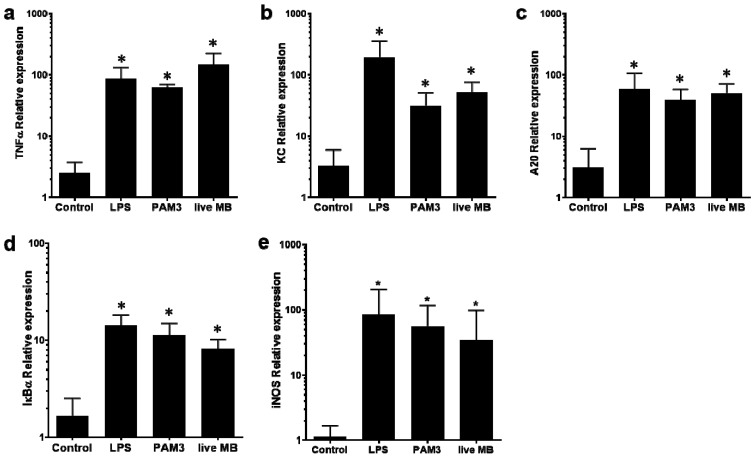
Gene expression of inflammatory mediators in murine mammary glands challenged with live *M. bovis*, LPS and PAM3. Gene expression was tested 24 h pi following IMM challenge using relative qRT-PCR. Bar graphs (mean and SD) show relative expression of TNFα (**a**), KC (**b**), A20 (**c**), IkBα (**d**) and iNOS (**e**) in control and challenged glands. * Indicates statistical significance in comparison with a control group (*p* < 0.05).

The ability of M. bovis to evoke an inflammatory response in mammary tissue following the IMM challenge was then demonstrated using qRT-PCR analysis (Figure 3). The results showed significant overexpression of TNF-α, KC, iNOS, A20, and IkBα genes following IMM challenge with *M. bovis* as well as with the MAMPs PAM3 and LPS used as positive controls (Figure 3).

### 3.3. Induction of Mammary Inflammation by LAMPs and M. bovis

**Figure 4:**
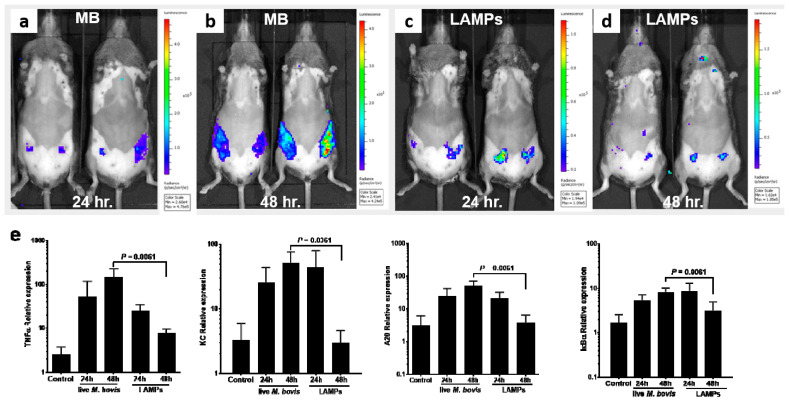
Temporal dynamics and spatial distribution of neutrophil recruitment and inflammation following IMM challenge with live *M. bovis* and *M. bovis*-LAMPs. Bioluminescence imaging of neutrophil recruitment was performed in mice challenged with live *M. bovis* (**a**,**b**) and LAMPs (**c**,**d**), 24 h (**a**,**c**) and 48 h (**b**,**d**) pi. Relative expression of TNFα, KC, A20, and IkBα genes 24 h and 48 h pi was analyzed using qRT-PCR (**e**).

Lipoproteins are the major MAMPs of mycoplasmas [59,60] and as we showed here, they elicit a significant inflammatory response in mMECs (Figure 1). To further demonstrate their role as important virulence factors of mammary pathogenic *M. bovis* and to investigate if there is a difference in the elucidation of inflammation between live *M. bovis* and LAMPs, mammary glands were challenged with either LAMPs or with live bacteria (Figure 4). The results showed that while LAMPs were sufficient to elicit mammary inflammation, important differences were unveiled in comparison with a bacterial challenge. Indeed, live *M. bovis* caused diffuse inflammation affecting the whole gland and peaking at 48 h pi (Figure 4a,b), while LAMPs elicited only focal inflammation, peaking at 24 h pi and resolving at 48 h pi (Figure 4c,d). These results were in concordance with qRT-PCR analysis of mammary tissues harvested at 24 h and 48 h following the challenge (Figure 4e). While the relative expression of the inflammation genes TNFα, KC, A20, and IkBα was similar for LAMPs and bacterial challenge at 24 h pi, their expression was significantly higher at 48 h pi, following bacterial challenge (Figure 4e).

## 4. Discussion

In the present study, *M. bovis* 161791 field strain was successfully used to establish and characterize in vitro murine MEC-based and in vivo murine mastitis model systems (Figure 1 and Figure 2). MECs play a major role in the innate immune system of mammary gland providing the first line of defense against invading pathogens and their MAMPs [61]. Interaction between MAMPs and PRRs, expressed on the host cells, leads to the activation of several signaling components, including NF-κB, which is a major regulator of inflammation in the mammary gland regulating more than 200 dependent genes, such as cytokines, chemokines, and other inflammatory mediators and modulators [36,62]. Activation of the NF-κB pathway by *M. bovis* and their LAMPs has been studied previously in different models in vitro [28,35,39,63], but not in mMECs. Our results revealed that live *M. bovis*, UV-treated *M. bovis*, and LAMPs are all potent activators of NF-κB in mMECs, but live *M. bovis* bacteria seem to show higher potency in comparison with UV-killed organisms (Figure 1). Interestingly, Gondaira et al. reported increased expression of immune genes in bMECs, stimulated with heat-inactivated *M*. *bovis*, but not with live cells concluding that live bacteria suppress the immune response [34]. In contrast, Zbinden et al. demonstrated that only live *M. bovis* triggered an immune response in bMEC suggesting that secreted secondary metabolites play an important role in the activation of the immune system [64]. In this study, both the *M. bovis*-live and *M. bovis*-UV-treated cells activate mMECs (Figure 1). We can speculate that UV-killed bacteria represent better the MAMPs activity of mycoplasma than heat-inactivated bacteria and that some of the MAMP ligands are temperature sensitive.

In addition, mammary virulence of *M. bovis*, characterized by intense neutrophil recruitment, loss of milk in the affected glands, and activation of major inflammatory mediators, was demonstrated using the murine mastitis model (Figure 2 and Figure 3). NF-kB-dependent genes such as A20 and IkBα, which form an internal regulatory loop of the NF-kB pathway [65], were upregulated following the challenge with *M. bovis* (Figure 3 and Figure 4). We also observed significant overexpression of TNF-α, KC (CXCL1), and iNOS (NOS2), all of which are major inflammatory mediators in the mammary gland activating massive recruitment of blood neutrophils into the alveolar and milk spaces (Figure 2 and Figure 4). In addition, CXCL1 is probably the most important component of the molecular mechanism enabling trans-endothelial migration of blood neutrophils en route to the alveoli [66]. Using a set of knock-out mice, we previously showed that neutrophil recruitment in response to LPS/TLR4 signaling was mediated by TNF-α, produced by alveolar macrophages, and was dependent on IL1-β and KC signaling and regulated by NO [41,42]. Pending further research, we can only speculate that similar inflammatory mechanisms are also activated by *M. bovis*.

Here, we show that LAMPs are sufficient to induce activation of inflammation by mMECs in vitro and inflammation and disease following IMM challenge; hence, should be regarded as virulence factors of mammary pathogenic *M. bovis*. Lipoproteins are extremely abundant in the cell membrane of *M. bovis*, and they are crucial for bacterial pathogenesis [60,67]. However, important spatial and temporal differences between mammary inflammatory response to challenge by live bacteria and LAMPs were identified in this study (Figure 4), suggesting that live pathogens express additional, not lipoprotein-related, virulence factors and/or secreted secondary metabolites, which might impact the host immunity and the progression and severity of the infectious process. Moreover, live mycoplasma organisms might be able to better disseminate in the tubular and alveolar milk systems and elicit widespread inflammation. It is also plausible that larger amounts of mycoplasmal LAMPs are necessary to stimulate diffuse and prolonged inflammation in lactating mice as we show here for live *M. bovis* (Figure 4a,b). Further research is required to better understand these differences and the experimental systems describes in this study should be useful to this end.

Attempts to establish a laboratory model in mice using *Mycoplasma* and *Ureaplasma* spp. have already been made in the past [68,69]. Anderson et al. performed IMM challenge study in mice with different *Mycoplasma* spp., including *M. bovis* and reported neutrophil infiltration and involution of inoculated glands [69]. However, these seminal studies were performed in genetically undefined and outbred conventionally kept mice at times when most of the currently used analytical tools were not available. Nevertheless, our in vivo results recapitulated these previously described studies in mice and are also consistent with clinical observations in field cases of the disease [11,70,71]. In addition, the murine model system enabled us to demonstrate *M. bovis* bacteria adherent to the apical membrane of mMECs, bacteria phagocytosed by luminal neutrophils and intraepithelial bacterial communities (Figure 2i,j). The interaction and colonization of *M. bovis* with MECs are vital for pathogen proliferation and pathogenesis, especially since its ability to replicate in milk is questionable [72], even though the shedding of *M. bovis* into the milk during mastitis is very high and may reach up to 10^9^ CFU/mL [73]. To date, the mechanisms by which *M. bovis* attach and invade the host cells are largely unknown. Recently, clathrin-dependent endocytosis was suggested as a pathway by which *M. bovis* invades synovial cells [74]. The internalization into host cells via an endocytic pathway has been previously shown for *Ureaplasma parvum* and *M. hyopneumoniae* [75,76]. Moreover, Nishiumi et al. suggested that transmission of *U. parvum* among cells is exosome-mediated [75]. Invading MECs as well as the formation of intraepithelial bacterial communities may help mammary pathogens to escape the host immune response and antibiotic treatment, and to establish persistent infection. We have previously demonstrated intraepithelial bacterial communities in coliform mastitis and suggested that bacterial survival in recruited neutrophils actively invading MECs is a possible mechanism of bacterial invasion [52].

Despite the existing differences between ruminants and murine mammary glands, the murine mastitis model is widely used to study the pathogenesis and control of bovine mastitis as well as for primary evaluation of new IMI antimicrobial compounds [77,78]. Mouse models offer several advantages such as time and cost effectivity, reproducibility, and the ability to reduce variability among animals by using genetically identical breeds. Moreover, the availability of genetically modified mice and multi-omic tools make the murine mastitis model an ideal in vivo experimental platform for the study of bovine mycoplasma mastitis [77,78,79].

## 5. Conclusions

The incapability of host immune response and antibiotics to prevent and combat *M. bovis* intra-mammary infection outlines the importance of future investigation of this major mastitis pathogen. Results presented in this study underscore the importance of virulence factors and/or secreted metabolites produced by live bacteria, in addition to their LAMPs, as innate-activators ligands and the importance of NF-kB activation by these ligands. The in vitro mMEC-based and in vivo *M. bovis* murine mastitis models developed and characterized in this study may help to evaluate *M. bovis* virulence factors and host immune response to IMI; both aspects are crucial in order to propose new prophylactic and therapeutic alternatives to counteract *M. bovis* mastitis.

## Data Availability

We declare that the data supporting the findings of this study are available within the paper.
